# Long-read sequencing of hundreds of diverse brains provides insight into the impact of structural variation on gene expression and DNA methylation

**DOI:** 10.1101/2024.12.16.628723

**Published:** 2024-12-17

**Authors:** Kimberley J. Billingsley, Melissa Meredith, Kensuke Daida, Pilar Alvarez Jerez, Shloka Negi, Laksh Malik, Rylee M. Genner, Abraham Moller, Xinchang Zheng, Sophia B. Gibson, Mira Mastoras, Breeana Baker, Cedric Kouam, Kimberly Paquette, Paige Jarreau, Mary B. Makarious, Anni Moore, Samantha Hong, Dan Vitale, Syed Shah, Jean Monlong, Caroline B. Pantazis, Mobin Asri, Kishwar Shafin, Paolo Carnevali, Stefano Marenco, Pavan Auluck, Ajeet Mandal, Karen H. Miga, Arang Rhie, Xylena Reed, Jinhui Ding, Mark R. Cookson, Mike Nalls, Andrew Singleton, Danny E. Miller, Mark Chaisson, Winston Timp, J.Raphael Gibbs, Adam M. Phillippy, Mikhail Kolmogorov, Miten Jain, Fritz J. Sedlazeck, Benedict Paten, Cornelis Blauwendraat

**Affiliations:** 1. Center for Alzheimer’s and Related Dementias, National Institute on Aging and National Institute of Neurological Disorders and Stroke, National Institutes of Health, Bethesda, MD, USA; 2. UC Santa Cruz Genomics Institute, Santa Cruz, CA, USA; 3. Laboratory of Neurogenetics, National Institute on Aging, National Institutes of Health, Bethesda, MD, USA; 4. Department of Neurodegenerative Disease, UCL Queen Square Institute of Neurology, University College London, London, UK; 5. Department of Biology, Johns Hopkins University, Baltimore, MD, USA; 6. Human Genome Sequencing Center, Baylor College of Medicine, Houston, TX, USA; 7. Department of Genome Sciences, University of Washington, Seattle, Washington, USA; 8. Division of Genetic Medicine, Department of Pediatrics, University of Washington, Seattle, Washington, USA; 9. DataTecnica, Washington, DC, USA; 10. Google LLC, Mountain View, CA, USA; 11. Human Brain Collection Core, Division of Intramural Research, National Institute of Mental Health, NIH, Bethesda, MD, USA; 12. Genome Informatics Section, Computational and Statistical Genomics Branch, National Human Genome Research Institute, National Institutes of Health, Bethesda, MD, USA; 13. Department of Pediatrics, Division of Genetic Medicine, University of Washington and Seattle Children’s Hospital, Seattle, WA, USA; 14. Department of Laboratory Medicine and Pathology, University of Washington, Seattle, WA; 15. Brotman Baty Institute for Precision Medicine, University of Washington, Seattle, WA.; 16. Department of Quantitative and Computational Biology, University of Southern California, Los Angeles, CA, USA; 17. Department of Biomedical Engineering, Johns Hopkins University, Baltimore, MD, USA; 18. Center for Cancer Research, National Cancer Institute, National Institutes of Health, USA.; 19. Department of Bioengineering, Department of Physics, Khoury College of Computer Sciences, Northeastern University, Boston, MA, USA; 20. Department of Molecular and Human Genetics, Baylor College of Medicine, TX, USA; 21. Department of Computer Science, Rice University, Houston, TX, USA

## Abstract

Structural variants (SVs) drive gene expression in the human brain and are causative of many neurological conditions. However, most existing genetic studies have been based on short-read sequencing methods, which capture fewer than half of the SVs present in any one individual. Long-read sequencing (LRS) enhances our ability to detect disease-associated and functionally relevant structural variants (SVs); however, its application in large-scale genomic studies has been limited by challenges in sample preparation and high costs. Here, we leverage a new scalable wet-lab protocol and computational pipeline for whole-genome Oxford Nanopore Technologies sequencing and apply it to neurologically normal control samples from the North American Brain Expression Consortium (NABEC) (European ancestry) and Human Brain Collection Core (HBCC) (African or African admixed ancestry) cohorts. Through this work, we present a publicly available long-read resource from 351 human brain samples (median N50: 27 Kbp and at an average depth of ~40x genome coverage). We discover approximately 234,905 SVs and produce locally phased assemblies that cover 95% of all protein-coding genes in GRCh38. Utilizing matched expression datasets for these samples, we apply quantitative trait locus (QTL) analyses and identify SVs that impact gene expression in post-mortem frontal cortex brain tissue. Further, we determine haplotype-specific methylation signatures at millions of CpGs and, with this data, identify cis-acting SVs. In summary, these results highlight that large-scale LRS can identify complex regulatory mechanisms in the brain that were inaccessible using previous approaches. We believe this new resource provides a critical step toward understanding the biological effects of genetic variation in the human brain.

Structural variants (SVs), including insertions, deletions, duplications, inversions, and complex rearrangements, represent significant contributors to genetic variation within the human genome. These variations can influence gene expression, regulatory elements, and genomic stability, playing a critical role in shaping the genetic architecture of the human brain. Through these mechanisms, SVs impact individual brain structure^1^, cognitive abilities, and susceptibility to a range of neurological disorders ^2,3^. Notably, SVs are implicated in the pathogenesis of several neurodegenerative diseases. For instance, duplications of the *APP* gene have been linked to early-onset Alzheimer’s disease (AD)^4^, while duplications and triplications of the *SNCA* gene contribute to Parkinson’s disease (PD) and dementia with Lewy bodies (DLB) ^5^. Similarly, transposable element insertions in the gene *TAF1* are causative of X-linked dystonia Parkinsonism ^6–8^, and *PRKN* (Parkin) gene rearrangements are a known cause of early onset PD^9–12^. These examples underscore the critical role of SVs in the etiology of neurodegenerative disease and highlight the need for comprehensive characterization of these variants to fully understand brain disorders.

Despite their importance, conventional short-read sequencing (SRS) methods, while useful for many applications, are limited in their ability to accurately detect and genotype SVs^13^. The short read lengths, typically only a few hundred base pairs, make it difficult to span larger SVs, especially those in the kilobase range. Additionally, SRS struggles to resolve variants within repetitive genomic regions, which hinders both the resolution of SVs and the accurate phasing of variants, leading SRS-based approaches to miss more than half of the SVs present in any one individual. Long-read sequencing (LRS) technologies address these limitations by providing longer reads that can more effectively span large SVs, resolve repetitive regions, and phase variants across much larger genomic distances. Recent studies, including our own, have shown that LRS significantly improves SV detection and variant phasing, even in challenging genomic regions^14,15^.

Beyond these technical limitations, the historical focus on European populations in genomic research has also significantly restricted our understanding of genetic variation in other ancestries. This bias has hindered the discovery of population-specific variants, which are essential for understanding traits such as disease susceptibility, drug metabolism, and gene regulation across different populations. For example, the power of diverse datasets was demonstrated when a population-specific *GBA1* variant associated with PD was identified in African or African admixed ancestry populations^16^. As research continues to reveal substantial differences in genetic risk factors across ancestries, the development of inclusive and comprehensive genomic resources becomes crucial for improving the accuracy of disease mapping^17^ and ensuring more equitable, globally relevant healthcare solutions.

In this study we generate high-quality long-read genomes from the neurologically normal brains of 351 individuals of European and African or African admixed ancestry. Through this effort, we discover 234,905 SVs and produce locally phased assemblies that cover 95% of all protein-coding genes in GRCh38. In addition, we apply quantitative trait locus (QTL) analyzes to identify SVs that impact gene expression and methylation in post-mortem brain tissue. We make this resource publicly available through AnVIL^18^ and Alzheimer’s Disease Workbench (ADWB) https://www.alzheimersdata.org/ad-workbench and believe this will provide a critical step toward understanding the biological effects of genetic variation in the human brain, as well as providing the first large set of matched, ancestrally diverse controls with both long- and short-read genomic data as well as short-read transcriptomic data.

## Results

### Genetic Ancestry and sequencing overview of brain samples across cohorts

**Figure 1:**
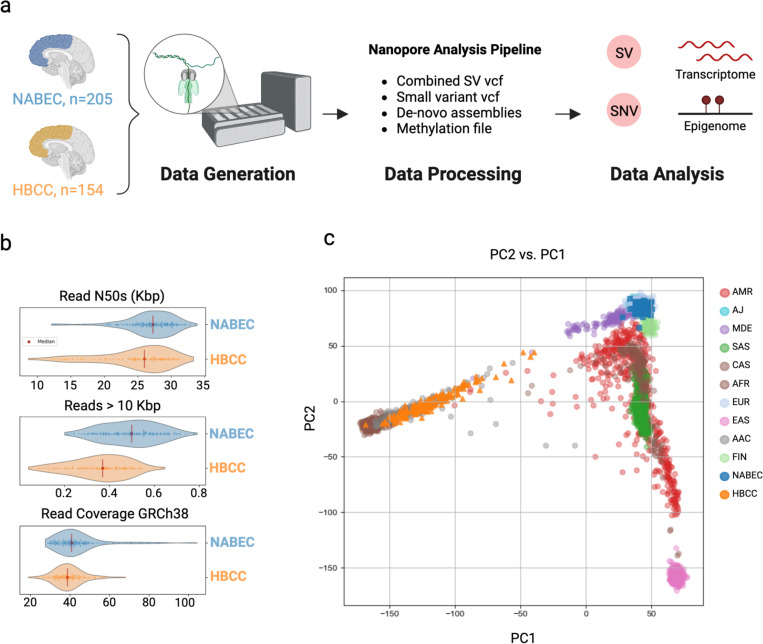
Overview of the study and samples. **a.** Graphical overview of the project workflow. **b.** Read N50s, proportion of reads longer than 10Kbp, and read coverage of GRCh38 for each cohort. Median values are denoted by the red vertical lines. **c**. Principal Component Analysis (PCA) of ancestry predictions using NABEC Illumina SNVs (blue squares) and HBCC ONT SNVs (orange triangles) overlaid on 1000 Genomes SNV ancestry clustering confirming that the NABEC cohort is predominantly of European ancestry while the HBCC cohort is of African or African admixed ancestry.

We conducted ONT whole-genome sequencing on 359 human brain samples from the NABEC (n=205) and HBCC (n=154) cohorts using the PromethION platform (Figure 1a). For the NABEC samples, sequenced on R9.4.1 flow cells using the LSK-110 library kit (referred to as R9), we obtained an average genome coverage of 46x and read N50 of 26.2 Kb. For the HBCC samples, sequenced on R10.4.1 flow cells and LSK-114 library kits (referred to as R10), we obtained an average genome coverage of 39x and read N50 of 24.6 Kb (Figure 1b). Full sequencing statistics are provided in Supplementary Table 1. To determine genetic ancestry, NABEC (Illumina) and HBCC (R10 ONT) SNVs were evaluated using data from the 1000 Genomes project, the Human Genome Diversity Project, and an Ashkenazi Jewish panel with GenoTools^19,20^. Predicted ancestry labels for each sample are provided in Supplementary Table 2. Comparative analysis with 1000 Genomes samples showed that, as expected, NABEC samples predominantly clustered with the European (EUR) group, while HBCC samples were more dispersed but predominantly clustered with the African ancestry cluster (Figure 1c). No individuals were excluded from the NABEC cohort, while eight individuals from the HBCC cohort, initially labeled as EUR, SAS, and AMR, were excluded from downstream analyses to reduce population structure and admixture effects in the QTL analysis. The final set of 351 samples was processed using the NAPU pipeline^14^, generating four key outputs for downstream analyses: de novo assemblies, harmonized SV calls from alignment- and assembly-based methods, alignment-based small variant calls, and haplotype-resolved methylation analysis.

### Generating Genome Assemblies at scale

**Figure 2:**
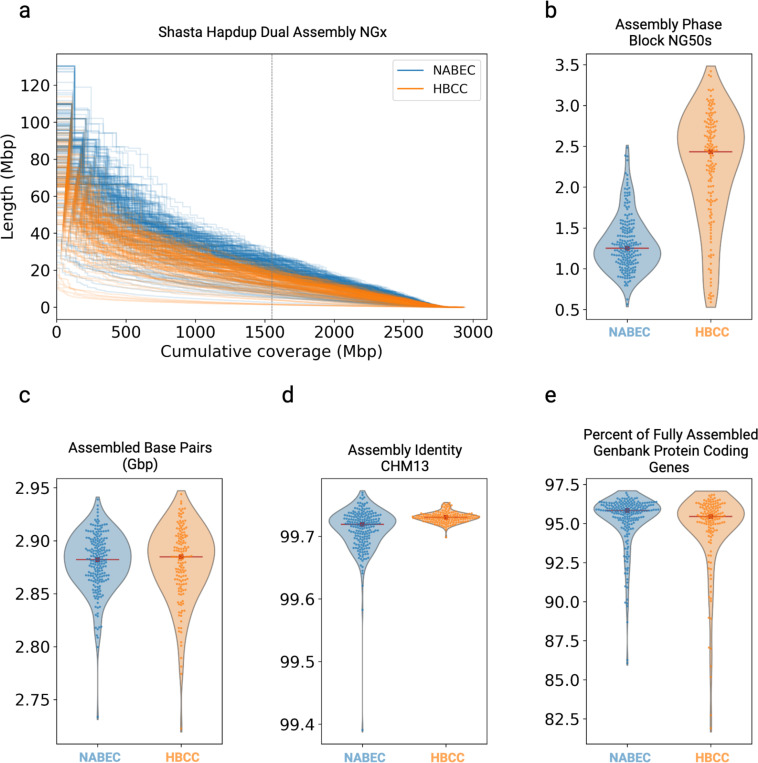
Assembly statistics for both cohorts **a.** Shasta+Hapdup Dual assembly NGx plot for HBCC and NABEC Cohorts, compared to the 3.1 Gb length of the T2T-CHM13v2.0 assembly. **b.** NG50’s of phased assemblies illustrate the increased phase block size of African American ancestry HBCC R10 samples. The red line is the median value. **c.** The number of assembled base pairs in dual assemblies aligned to CHM13v2.0. **d.** Contig identity mapped to CHM13. **e.** Percentages of Genbank v44 protein-coding genes completely assembled in each samples’ dual assembly.

All samples were assembled with Shasta^21^ and phased into diploid assemblies with Hapdup^14^. Hapdup phases Shasta assembly contigs using the ONT reads to produce locally phased ‘dual’ and phased assemblies, in which contigs are the phase blocks. Shasta was run with an R9 and R10 ONT configuration for the NABEC and HBCC cohorts respectively. NG50s are lower for HBCC dual assemblies but, due to the genetic heterozygosity of the African or African admixed HBCC samples, we did observe longer phase blocks for HBCC assemblies than the European ancestry NABEC samples (Figure 2a, 2b). The NABEC cohort dual assemblies had a median NG50 of 21.4 megabases (Mb) while the median NG50 for HBCC dual assemblies’ is 15.7 Mb (measured against the 3.1 gigabase (Gb) length of the T2T-CHM13v2.0 assembly). The phase block median NG50 is 1.25 Mb and 2.43 Mb for NABEC and HBCC, respectively. Despite these differences, samples from both cohorts assembled 94% of GRCh38 with a median 2.88 Gb assembly size (Figure 2c). Importantly we found that Shasta assembled 99% of the regions labeled as “difficult” in GRCh38 by the Genome In a Bottle Consortium (GIAB)^22^. To measure assembly quality we estimated the base level accuracy by alignment to the more complete CHM13 reference and computed the median divergence from the reference of 0.26–0.28% (contig identities to be 0.9971 and 0.9973) for NABEC and HBCC, respectively (Figure 2d).

Next, we assessed the quality and usefulness of the assemblies’ gene models. The 62,700 Genbank v44 genes were assessed for assembly completeness; across all samples, the median number of completely assembled genes is 58,566 (93.4%); mean 58,337, (93%). The median number of protein-coding genes assembled completely in both haplotypes across all samples is 19,181 (95.6%); mean 19,048, (95.0%) (Figure 2e). We examined 168 genes identified as associated with PD or AD in previous GWAS studies ^23,24^ (Supplementary Table 3). On average, 160 of the 168 GWAS genes (95%) are assembled contiguously. The majority of the genes reported as not completely assembled are partially assembled in one or both of the assembly haplotypes. The 174 immunoglobulin heavy locus (IGH) genes in Genbank have also been associated with AD and PD disease risk; we assessed assembly of these and found 97% (169 median) of Chr14 IGH genes are assembled over all the samples. Assembling the IGH loci in these 351 primary tissue samples is an important contribution of our work, since much of the large scale long read sequencing projects have used lymphoblast cell lines.

### Structural variant characterization across diverse cohorts

**Figure 3:**
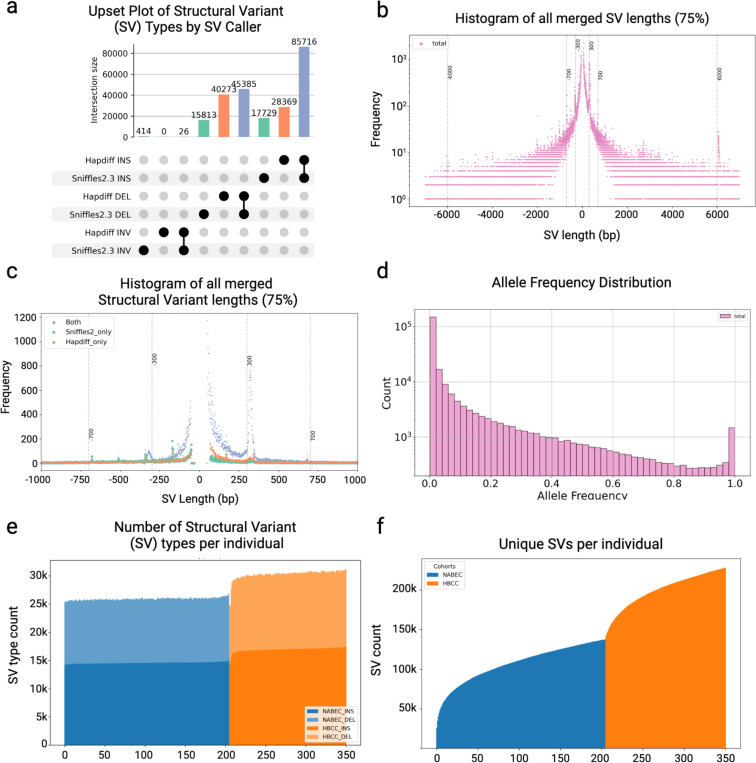
Structural variant characterization across both cohorts **a.** An upset plot of SVs by SV caller and SV type tallied after merging. Hapdiff called 28,369 insertions and 40,273 insertions that weren’t merged with Sniffles. Sniffles called 17,729 insertions and 15,813 deletions that weren’t merged with Hapdiff. 85,716 insertions and 45,385 deletions were called by both callers and merged together by Truvari. **b.** The size of insertions and deletions is plotted as negative for deletions and positive for insertions. Expected peaks of common SVs are shown at 300 bps, Alu repeats, 700 bps, SINE, and 6000 bps LINE1. **c.** Size of SVs that are merged between SV callers, shown in lavender, and called by just one SV caller. Sniffles SVs that weren’t merged with Hapdiff SVs are shown in green and Hapdiff only SVs are in orange. **d.** Allele frequency distribution of merged SVs. **e.** The number of SVs per individual is stratified by insertions in dark blue, NABEC, or dark orange, HBCC, and deletions in the lighter blue and orange. **f.** A cumulative bar chart that shows the number of new unique SVs added by each individual across both cohorts. The European NABEC cohort contributed ~115,000 SVs and the HBCC cohort contributed another ~125,000 SVs to create the set of 234,905 SVs merged across both cohorts and SV callers.

We identified SVs using two complementary approaches: Sniffles^15^, an alignment-based method, and Hapdiff^14^, an assembly-based method. To generate a unified set of SV calls, we merged the outputs of both tools using Truvari^25^, resulting in a final harmonized, union VCF. The majority of SVs (73% of SVs with allele frequency > 0.01) were detected by both Sniffles and Hapdiff (Figure 3a). SVs of expected lengths, like the 300 bp *Alu* insertion and 6000 bp *LINE* insertion peaks, were called by both SV callers (Figure 3b). However, after merging some SVs were only identified by one caller. In particular, Hapdiff seems to call more smaller insertions than Sniffles (Figure 3c). Most SVs were rare, with 59% having a minor allele frequency (MAF) below 1% (Figure 3d).

In the NABEC cohort, we detected on average 25,937 SVs per genome (Figure 3e) which on average impacted 15.4 Mb of inserted genomic sequences and 7.4 Mb of deleted sequence per individual. As expected due to its more diverse ancestry^26^, more SVs were detected in the HBCC cohort, with an average of 30,322 high-confidence SVs per genome and on average impacted 15.7 Mb of inserted sequence and 7.9 Mb of deleted genomic sequence per individual (Figure 3e). Most SVs per sample were insertions (average 15,556 (55.98%) combined per individual) and deletions (12,204 (43.89%) combined per individual), with inversions less frequent (31 inversions on average (0.12%)). These results are consistent with findings from other large-scale long-read sequencing studies, such as the 1000 Genomes Project^27^, which reported an average of 24,543 SVs per genome,the Human Genome Structural Variation Consortium (HGSVC^26^), which reported an average of 27,622 SVs per genome, the DECODE Icelandic study with 22,636 SVs per genome^28^, and the NIH All of Us study, which identified 24,285 SVs per genome^29^.

Across both cohorts, we identified 234,905 high-confidence SVs, consisting of 131,813 insertions, 102,651 deletions, 440 inversions. Among the 90,580 SVs that intersect protein-coding genes, 93.1% (84,296) were located in intronic regions, 6.9% (6,234) overlapped coding regions, and 4.1% (3,715) were found within or spanned the 5’ or 3’ untranslated regions (UTRs). The average SV size was 3,681 bp, ranging from 50 bp to 76 Kbp. As observed in previous studies, insertion sizes peaked at approximately 300 bp and 6 kb, corresponding to *Alu* and LINE1 transposable elements, respectively ^27,30^. We annotated the SVs with known clinical SV databases (nstd102 in dbVar) or any DGV SVs (GRCh38_hg38_variants_2020–02-25.txt) using sveval^31,32^ and as expected no known pathogenic SVs were detected in the healthy controls. We next assessed the population allele frequencies of SVs identified in our study by comparing them to frequencies reported in 1,108 long-read genomes from the 1000 Genomes Project and the Human Pangenome Reference Consortium (HPRC) using the STIX tool^33^. A significant linear correlation was observed when comparing allele frequencies from NABEC to EUR STIX frequencies and HBCC to AFR STIX frequencies, indicating strong concordance between our data and these reference datasets (Supplementary Figure 1). To estimate the proportion of novel SVs, defined as variants not reported in previous large-scale long-read sequencing studies, we also utilized the STIX tool. Our analysis revealed that 32.5% of SVs across the two cohorts were novel.

### The impact of structural variants on gene expression in the human frontal cortex

We performed an SV-only QTL analysis to evaluate the overall impact of SVs on gene expression in the human frontal cortex. Focusing on common variants (MAF > 5% within this cohort), we investigated their cis-regulatory effects using gene-level whole-transcriptome data from short-read RNA-seq.^34–36^ Expression data was available for 205 of the NABEC samples, and 76 of the HBCC samples. After filtering 24,375 SVs were included in the eQTL analysis, with expression data including 25,887 expressed genes, of which 15,502 were protein-coding. In the HBCC, 38,018 SVs remained, with expression data including 25,847 expressed genes, including 15,401 protein-coding genes. An eQTL was defined as an eVariant/eGene pairs, with a *cis* window that included variants located within 1 Mb of the gene’s transcriptional start site (TSS).

Using TensorQTL and applying a standard false discovery rate (FDR) threshold of q-value < 0.05, we identified 838 significant SV-eQTL (q-value <0.05) in the NABEC datasets affecting 779 distinct eGenes and 135 significant SV-eQTL in the HBCC affecting 134 distinct eGenes (Table 1). In terms of overlap, 55 (NABEC: HBCC ; 7.1% : 41.0%) of the significant SV-eQTL were common across both cohorts. To gain insight into the genotyping accuracy of the SV calls we manually reviewed 30 randomly selected significant SV-QTL. Through this we manually validated 26/30 SV (86.7%) in NABEC and 23/30 (76.7%) in HBCC by corresponding changes in read coverage using IGV. We observed a bimodal distribution of effect directions (β) across SV types for non-coding SV-eQTL. Consistent with previous studies, when focusing on SV-eQTL that overlap exons, we find that deletion SVs are generally associated with decreased gene expression ^37^(Supplementary Figure 2). Similarly, inline with short-read-based SV-QTL studies, we observe that many insertion SV-eQTL result in increased gene expression when the insertion overlaps an exon (Supplementary Figure 2).

To further assess the contribution of SVs to gene expression in the frontal cortex, we performed a joint eQTL analysis combining SVs and small variants (SNVs and indels). Due to the lower SNV calling accuracy of ONT R9 compared to ONT R10, we used Illumina-based SNV data for NABEC and ONT-based SNV data for HBCC. In the NABEC, we identified 2,208 significant eQTL affecting 2,064 distinct eGenes , 49 of which had an SV as the lead variant. In HBCC, we identified 1,165 eQTL affecting 1,142 distinct eGenes , 16 of which had an SV as the lead variant (Table 1). Notably, 391 eQTL were shared between the cohorts (NABEC: HBCC; 18.9%, 34.2%), which aligns with previous SNV-based QTL studies reporting approximately 30% overlap in eQTL across cohorts of European and African admixed ancestry^38^. The limited overlap may be influenced by the difference in sample number between the NABEC and HBCC cohorts, given that the HBCC cohort includes short-read RNA-seq data for only 76 samples, in contrast to 205 samples in NABEC.

### SVs as top candidate variants driving eQTL associations in the human frontal cortex

To assess the potential causal role of SVs at each locus, we performed statistical fine-mapping using CAVIAR, estimating the posterior probability of each SV being causal compared to nearby small variants within a 1 Mbp window, while accounting for linkage disequilibrium (LD) between variants. For each of the 2,064 eQTLs in NABEC and 1142 eQTLs in HBCC, we identified the top 100 variants within a 1 Mb cis window that were most significantly associated with the eGene, ranking variants based on their p-values. CAVIAR was then used to assign a causal likelihood and relative ranking to each of these 100 variants based on the strength and direction of their associations, as well as the pairwise LD structure across the region^39^. Of the 2,064 loci analyzed in NABEC, 2052 contained both an SV and small variants for testing, while 12 regions contained no SV, so only small variants were tested. Of the 1,142 loci analyzed in HBCC, 1,126 contained both an SV and small variants for testing, while 16 regions contained no SV, so only small variants were tested. In the NABEC cohort, SVs were identified as the most likely causal variant in 2.9% (59/2064) of eQTL, while in the HBCC cohort, SVs were the top probable causal variant in 1.4% (16/1142) of eQTL (Table 1). These results align with findings from Kirsche et al., who also applied long-read-derived SV-eQTL fine-mapping and reported high posterior probabilities for SVs in 3.65% of eGenes. This work suggests that many previously undetected SVs may play a significant role in differential gene expression, compared to the SNVs identified in earlier eQTL studies^40^.

**Figure 4:**
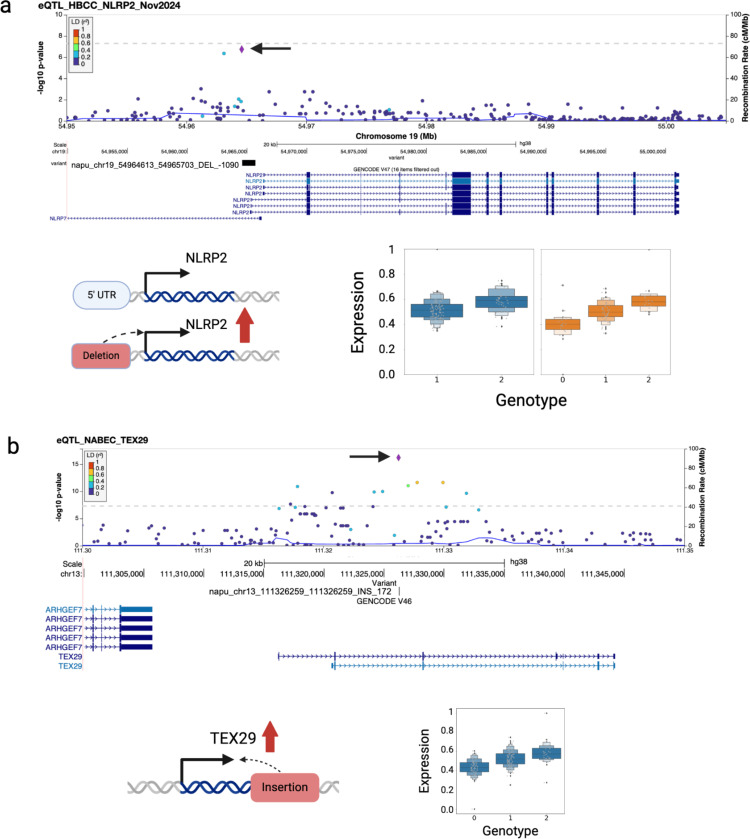
eQTL discovery and fine-mapping **a.** Example of an eQTL in the NLRP2 gene. The top panel presents a locuszoom plot of the SV and small variant joint eQTL for HBCC, with an arrow indicating the top significant variant, napu_chr19_54964613_54965703_DEL_−1090. The middle panel illustrates a deletion overlapping the 5’ transcription start sites of several transcripts of NLRP2. The bottom left panel shows a boxplot of the eQTL stratified by genotypes of the deletion (NABEC in blue, HBCC in orange). The bottom right panel depicts a schematic representation of the effect of the variant. **b.** Example of an SV and small variant joint eQTL driven by an SV in NABEC. The top panel presents a locus zoom plot of the SV and small variant joint eQTL for NABEC, with an arrow indicating the top significant variant, napu_chr13_111326259_111326259_INS_172. The middle panel shows the variant overlapping an intron of *TEX29*. The bottom left panel displays a boxplot of the eQTL stratified by genotypes of the insertion (NABEC in blue). The bottom right panel depicts a schematic representation of the variant’s effect.

Among the 55 candidate causal SVs identified across both cohorts, one notable example is located at the *NLRP2* locus. *NLRP2*, part of the NLR family of immune-related genes, plays a key role in regulating inflammatory responses^41^. A 1,090 bp deletion (napu_chr19_54964613_54965703_DEL_−1090) in the 5’ untranscribed region (UTR) of *NLRP2* is associated with reduced gene expression in the frontal cortex within the HBCC cohort (nominal p-value = 8.78 × 10^−7^) (Figure 4a). However in the NABEC cohort, this deletion was excluded from the QTL analysis due to a low genotyping rate caused by challenges in SV merging. Instead, another SV tagging the deletion, a 1,382 bp insertion (napu_chr19_54965640_54965640_INS_1382) was identified as the candidate causal variant. This insertion was in high LD with the deletion (R² = 0.97, D’ = 1). Another example, that was only significant in the NABEC cohort, is an 172 bp insertion (napu_chr13_111326259_111326259_INS_172) located within the gene *TEX29* (Testis Expressed 29). This insertion was associated with increased gene expression in carriers (NABEC p-value = 5.96 × 10^−17^) (Figure 4b).

### Genome-wide Methylation profiling in the human frontal cortex

**Figure 5:**
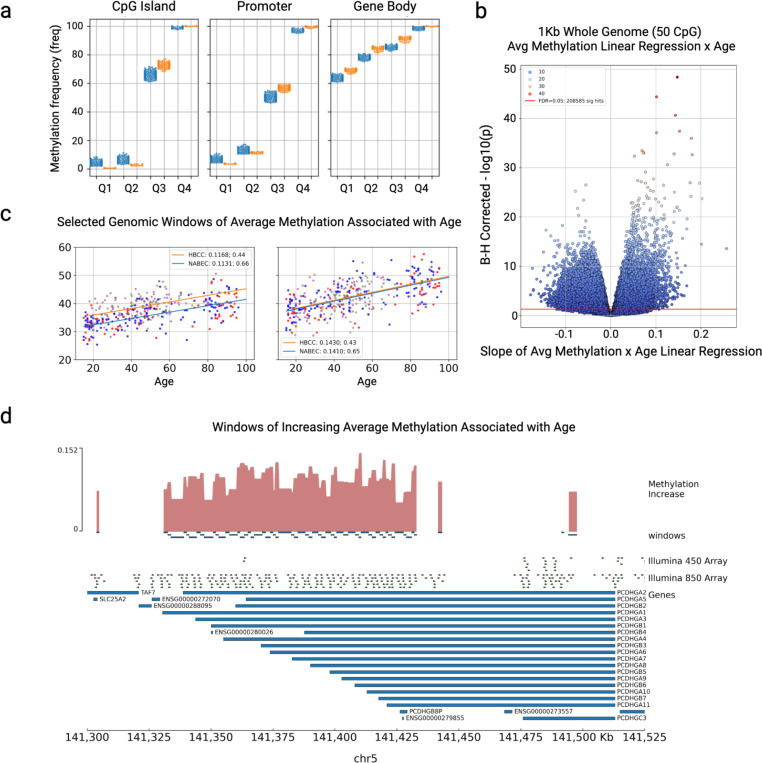
Profiling genome-wide methylation in the frontal cortex **a.** Aggregated methylation frequency of NABEC (blue) and HBCC (orange) samples by quartile for CpG islands, promoter regions ( extended to 2kb ), and across RefSeq gene bodies. **b.** Age associations of whole genome 1Kb / 50 CpG minimum windows of averaged methylation in the NABEC cohort. Many regions (145,086; FDR 0.05) were weakly associated with age; the slope of the linear regression ranged from −0.1 to 0.1. The volcano plot is of age regressions; x-axis is the slope of the regression line y-axis is the Benjamini-Hochberg corrected p-values. **c.** Two neighboring regions significantly associated with age that overlap exons of the Protocadherins Gamma (PCDHAG) cluster of genes. **d.** Tracks of windows showing methylation increase with age. Top track is a barplot of the amount of methylation increase (slope) by age. Window locations associated with age are shown below. The locations of Illumina 450 and EPIC 850 Methylation Arrays are shown above the gene locations. Across the bottom are coordinates of Chr 5.

LRS enables the simultaneous detection of both sequence variation and DNA methylation directly from native DNA. LRS-based methods provide more complete and contiguous methylation profiles than short-read technologies, which often rely on indirect methods like bisulfite conversion and can miss key regulatory regions due to fragmented or incomplete coverage^42^. Prior work has shown that methylation calls in brain tissue samples reflect the expected distribution of methylation patterns of primary tissue, and in this case, prefrontal cortex tissue specifically^43^. The majority of CpG’s are hypermethylated (>60% methylation frequency) in these tissues and less than 10% of whole genome windows are hypomethylated (<60% methylation frequency). Methylation was aggregated across the genome by averaging over windows of at least 1 Kbp and expanding the window to include a minimum of 50 CpGs. The resulting sizes of these windows range from 1Kb to over 10Kbp although only 10% are 10Kbp or greater. Methylation calls in R10 samples are more often closer to 0 or 100% methylation when compared to R9 methylation calls. The technical differences in R9 and R10 methylation measurements are apparent in Figures 5a (Supplementary figure 3)^44^. Previous work has shown that R10 is more accurate than R9 when compared to Whole Genome Bisulfite Sequencing^44^. Aggregating methylation calls, however, can minimize this difference. Due to this difference, we analyzed the samples in the NABEC and HBCC cohorts separately.

Using the 1Kb windows and CpG islands (CGIs) we looked for regions whose methylation status is correlated with age. To do this we performed an ordinary least squares linear regression with age as the independent variable and average methylation frequency as the dependent variable. The NABEC cohort has 51 more individuals and longer-lived individuals on average than the HBCC cohort thus it has stronger correlations with age than HBCC. Moreover, the admixture of the African American samples may contribute to the smaller number of correlations with age^45^. Around 28% of the 518,000 whole genome windows and 14% of the CGIs in the NABEC cohort were significantly (FDR < 0.05) associated with age. Similarly in HBCC 8.5% of the 518,466 coverage filtered windows and 14% of the CGIs in the HBCC cohort were significantly (FDR < 0.05) associated with age (Figure 5b). When comparing these results across cohorts 5% of the genome windows and 1% of the CGIs showed significance in both cohorts and in the same direction (Supplementary Table S21). For most CGIs and genome windows, the change in methylation represented by the regression slope was subtle (less than 0.1). However, when looking at the CGIs and 1Kb windows with the largest methylation changes (slopes greater than 0.1 and an R^2^ of more than 0.4) they do overlap with some functionally relevant regions. For example, many regions significantly associated with age overlap the protocadherin alpha and gamma clusters of genes on the q-arm of chromosome 5 (Figure 5c, 5d). Genes from these clusters create protein products that combine to produce unique protein markers on the outside of each neuron in the human brain responsible for distinguishing a neighboring neuron from itself.^46^ Fewer regions had a decrease in methylation with age, such as the *FKBP5* gene, which functions as mediator of glucocorticoid receptor cortisol binding and signaling^47^, and had the largest decrease in methylation frequency by age. *FKBP5* binding to glucocorticoid receptors is mediated by its epigenetic status and ultimately can prolong the body’s stress response through its impact on the levels of circulating cortisol and glucocorticoids^47,48^. Interestingly overexpression of *FKBP5* may also play a role in *Tau* protein accumulation which is linked to AD^48^.

### Structural Variants Influence DNA Methylation in the human Frontal Cortex

Genetic variation plays a critical role in shaping DNA methylation patterns in the human brain, influencing gene regulation and contributing to neurodevelopment and disease complexity ^49,50^. While small variants have been well studied in this context, the influence of SVs remains less understood due to challenges in detection and genotyping. Building on the eQTL framework, we performed mQTL analyzes to evaluate the effects of SVs on DNA methylation in the postmortem frontal cortex, focusing on CGIs (defined as the 27,949 CpG Islands in GRCh38, with more than twice the expected number of CG dinucleotides based on GC content of the rest of the genome), promoter regions, and gene bodies. ^51^. We defined mQTL as cis-acting variants that significantly influence DNA methylation within a 1 Mb window surrounding each CGI, promoter, or gene body. Hence in this context, cis-acting refers specifically to variants affecting any methylation within this 1 Mb range; we did not assess haplotype-specific methylation in this study. Hereafter, we refer to these as CGI-mQTL, prom-mQTL, and gene-mQTL, respectively.

We first conducted an SV-only mQTL analysis. In the NABEC cohort, we identified 612 SV-CGI-mQTL across 567 distinct CGIs. Similarly, in the HBCC cohort 266 SV-CGI-mQTL were identified across 259 distinct CGIs. Of these, 100 (NABEC: HBCC ; 17.6%, 38.6%) were shared between both cohorts, exhibiting the same phenotypes and consistent directions of effect (β). Next, we examined promoter regions, identifying 652 SV-prom-mQTL in NABEC across 614 distinct promoters and 486 SV-prom-mQTL across 472 distinct promoters in HBCC (Table 2). A total of 117 (NABEC: HBCC ; 19.1%, 24.9%) of these were common across both cohorts, with the same SVs and direction of effect (β). The overlap observed here is smaller than that seen between eQTL hits across both cohorts, and may be due to greater variability in the methylation data compared to the expression data, as NABEC samples were sequenced on R9, while HBCC samples were sequenced on R10. This variability in R9 methylation data compared to R10 has been documented using both chemistries on the benchmark sample HG002 and may be contributing to the lack of overlap in eQTL hits, especially considering the change in methylation is often quite subtle^44^. For gene bodies, we identified 1687 SV-gene-mQTL in NABEC for 1581 distinct genes and 658 SV-gene-mQTL in HBCC for 682 distinct genes (Table 2), with 252 (NABEC: HBCC ; 16.0%, 37.0%) common between both cohorts, showing consistent SVs and effects (β). Similar to the SV-eQTL analysis, we observed a bimodal distribution of effect directions (β) between SV types for non-coding SV-mQTL at CGIs, promoter, and gene levels. Notably, when focusing on SV-mQTL overlapping exons, SV-prom-mQTL displayed a distinct pattern: insertions were generally associated with hypermethylation, while deletions were linked to hypomethylation (Supplementary figure 4).

Next to further assess the relative contribution of SVs to methylation in the postmortem frontal cortex, we conducted a joint QTL analysis combining SVs and small variants(Table 2). For the CGI-mQTL, prom-mQTL, and gene-mQTL, 419 (NABEC: HBCC ; 25.8%, 41.4%), 676 (NABEC: HBCC ; 33.4%, 30.2%), and 809 (NABEC:HBCC ; 20.1% : 40.0%) were shared between both cohorts, with the same phenotype and direction of effect (β). Overall, the odds ratio for an SV to be mQTL compared to small variants was 9.9 (95% CI: 8.16–11.91), 4.3 (95% CI: 3.44–5.48), and 6.5 (95% CI 5.54–7.55) for CGI-mQTL, prom-mQTL, and gene-mQTL, respectively, across both datasets, suggesting that individual SVs are more likely than individual small variants to act as an mQTL.

### SVs are amongst the top candidate variants driving mQTL associations in the human frontal cortex

**Figure 6:**
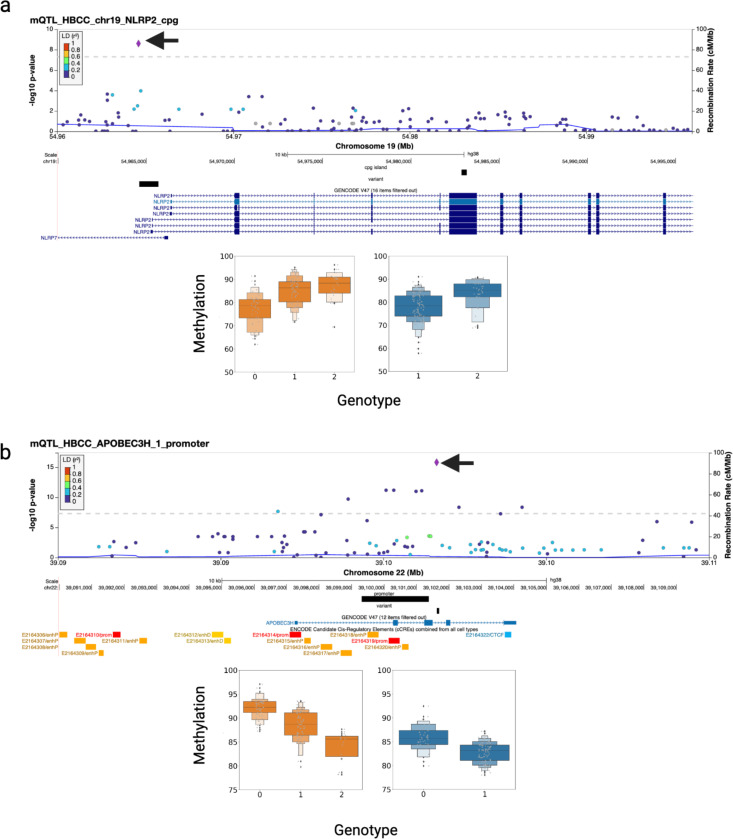
mQTL discovery and fine-mapping a. Example of a SV-SNV joint cpg-islands-mQTL led by a SV in the gene *NLRP2*. The top panel presents a locuszoom plot of the SV-SNV joint cpg-islands mQTL for HBCC, with an arrow indicating the top significant variant, napu_chr19_54964613_54965703_DEL_−1090. The middle panel illustrates the variant overlapping the 5’ transcription start sites of several transcripts of NLRP2 and the phenotype cpg island overlapping with the exon of *NLRP2*. The bottom panel shows a boxplot of the eQTL stratified by genotypes of the deletion (NABEC in blue, HBCC in orange). b. Example of a SV-SNV joint promoter-mQTL led by a SV in the gene *APOBEC3H*. The top panel presents a locuszoom plot of the SV-SNV joint cpg-islands mQTL for HBCC, with an arrow indicating the top significant variant, napu_chr22_39101634_39101696_DEL_−62. The middle panel illustrates the variant overlapping the intron of *APOBEC3H* and the phenotype cpg island overlapping with the exons and intron of *APOBEC3H*. The bottom panel shows a boxplot of the mQTL stratified by genotypes of the deletion (NABEC in blue, HBCC in orange).

To further assess the potential causal role of SVs at each locus, we performed statistical fine-mapping using CAVIAR. In the NABEC cohort, SVs were identified as the most likely causal variant in 4.2% (68/1623) of CGI-mQTL, 2.8% (56/2021) of prom-mQTL, and 4.2% (167/4026) of gene-mQTL. In the HBCC cohort, SVs were the top probable causal variant in 3.7% (37/1012) of CGI-mQTL, 2.0% (44/2236) of prom-mQTL, and 3.9% (78/2023) of gene-mQTL (Table 2). Across both cohorts, we identified 105 candidate causal SV-CGI-mQTL, 100 SV-prom-mQTL, and 245 SV-gene-mQTL. Among the 450 candidate causal SV-mQTL identified across both cohorts, one notable example is also a SV-eQTL located at the NLRP2 locus. We identified that the same 1090 bp deletion (napu_chr19_54964613_54965703_DEL_−1090) that overlaps the 5’ UTR of the *NLRP2* gene as the top candidate causal variant at this locus (Figure 6a). This deletion is significantly associated with hypermethylation in the frontal cortex of a CGI (chr19_54982861_54983167_CGI :_30) that overlaps with exon 6 in the HBCC cohort (p-value = 3.47×10^−16^). This observation is notable because hypermethylation is generally expected to correlate with transcriptional repression. However, in this case, the data suggest a more nuanced regulatory mechanism. Additionally, we identified a 62 bp deletion (napu_chr22_39101634_39101696_DEL_−62) within an intron of *APOBEC3H* as the top candidate causal variant at the *APOBEC3H* locus. *APOBEC3H*, a member of the *APOBEC3* family of cytidine deaminases, plays a crucial role in innate immunity by restricting viral replication, particularly retroviruses, through DNA deamination. This 62 bp deletion is associated (p-value = 1.04×10^−28^) with hypomethylation of an alternative promoter (chr22_39099320_39101380_APOBEC3H_1) in the frontal cortex, further supporting its role as a candidate causal variant (Figure 6b).

## Discussion

Long-read sequencing has significantly improved our ability to capture complex genetic variation, such as SVs, phase variants of interest, and provide detailed methylation data. SVs, in particular, have been shown to influence gene expression in the human brain and are linked to various neurological conditions, but their impact on methylation has been less studied. This highlights the critical role of long-read sequencing in uncovering the full spectrum of genetic contributions to brain function and disease, as it enables the detection of a broader range of genetic variants than traditional genotyping approaches ^52^. In this study, we present the first brain tissue based comprehensive long-read genomic resource derived from 351 human frontal cortex samples, offering a detailed catalog of 234,905 SVs, 34,087,867 small variants, and DNA methylation profiles across >28 million individual CpG sites. By integrating this dataset with matched short-read RNA sequencing data, we identified numerous SVs as top candidate causal variants influencing both gene expression and DNA methylation in the human brain. These findings provide new insights into the genetic and epigenetic architecture of the brain, demonstrating the power of long-read sequencing to reveal complex regulatory mechanisms. Importantly all data have been made accessible through cloud based analysis platforms to support further research into the functional role of SVs in neurodevelopment and neurodegeneration.

We produced highly contiguous and near-complete locally phased Shasta-Hapdup assemblies for 361 samples covering all but 6% of the GRCh38 reference. Although these assemblies are more fragmented than the multi-technology Human Pangenome Reference Consortium (HPRC) assemblies which have an average phased NG50 of 40Mb, we are able to produce these at scale in a fraction of the time using only one data type^14,53^. When compared to ONT sequencing and Shasta Hapdup assembly of 1000 Genomes Project (1KG) cell lines our assemblies of primary tissue from patients are as complete (93% complete; ~22–35Mbp NG50s to GRCh38) and nearly as contiguous^27^. The assemblies have high base level accuracy, and although highly identical duplicated genes are a challenge for Shasta, we were able to assemble 96% of genes which facilitated the calling of assembly-based SVs ranging from 50bp - 1Mbp. The assembly phase blocks are low, in comparison to HPRC fully phased assemblies, which is a current limitation of phasing without trio information, though as the length and quality of ONT reads increase we expect this to increase.

We demonstrate the power of integrating SV data with SNVs, where joint-QTL analysis and fine-mapping identified numerous SVs as likely causal variants influencing gene expression and methylation differences in the brain, variants that would have been overlooked using SRS. By overlapping methylation and expression results, we dissected complex regulatory mechanisms, exemplified by the *NLRP7/NLRP2* locus. At this region, a 1,090 bp deletion of the *NLRP2* promoter, flanked by two *Alu* transposable elements, emerged as a top hit in both mQTL and eQTL analysis. This deletion was correlated with increased *NLRP2* expression in the brain and hypermethylation of a CpG site within exon 6. Interestingly, these findings challenge the conventional view that promoter hypermethylation universally suppresses transcription. In this case, methylation appears to facilitate gene expression, likely through mechanisms involving chromatin structure, allele-specific methylation, or enhancer-promoter interactions. This underscores the context-specific regulatory roles of methylation and highlights the value of integrative analyses for uncovering atypical but biologically significant relationships. To assess the effectiveness of LRS in identifying causal SVs, we compared QTL results from LRS with those from SRS in the NABEC cohort. LRS identified more than twice the number of SV-associated QTLs compared to SRS (740 vs. 358; Supplementary Table 23). For example, the 1,090 bp deletion at the *NLRP7/NLRP2* locus, which LRS accurately resolved, was linked to changes in gene expression and DNA methylation but was undetectable using SRS. Similarly, an insertion associated with *TEX29* expression changes was identified through LRS but missed by SRS. These results demonstrate the superior ability of LRS to resolve complex genomic variations, advancing our understanding of the genetic and epigenetic mechanisms underlying brain function and disease.

However, it is important to acknowledge that despite the advantages of long-read sequencing, SV genotyping accuracy remains lower than SNVs. This is partly due to the technical and biological challenges in merging SV calls across different callers and samples. As a result, we believe the number of SVs identified as causal variants in fine-mapping analyses may be underestimated i.e. if an SV is genotyped less accurately than the small variants included in the testing, or if the SV is inherently variable such that its different suballeles are not merged, it could be excluded even if it is the true causal variant. Additionally, the eQTL analyzes in this study were based on short-read RNA sequencing data, which is often limited to gene-level resolution. Given that SVs can drive differential transcript-level expression and even generate novel transcripts, fully capturing their functional impact will require large-scale long-read RNA datasets which is the natural next step.

To our knowledge, this is the first large-scale analysis of brain tissue based methyl-QTL effort using LRS to assess the impact of SVs and the largest compilation of methylation data from primary brain tissue using LRS. It has been shown that genetic variation drives the correlation between methylation and gene expression^54^. To explore this connection we have taken advantage of all the data produced by the NAPU pipeline and performed SV/small variant mQTL analyzes in the brain to understand how genomic variants and epigenetic regulation drive gene expression. ONT-based methylation analysis has been proposed as a diagnostic tool for use in brain tumor sequencing and classification and for understanding disease progression using mouse brain tissue, and we suggest that our data will help facilitate these and other diagnostic and treatment efforts ^55,56^. We next plan to perform phased/allele specific mQTL analysis to determine cis-acting haplotype specific effects. We also expect SV and methylation genotyping to further improve as the necessary tools continue to develop.

We were able to correlate methylation signals with age across samples. It has been shown that changes in DNA methylation have been correlated with age and we were able to recapitulate many of these results which have mostly been identified using targeted arrays and bisulfite sequencing ^57,58^. ONT methylation data is on par with the quality and coverage of whole genome bisulfite sequencing, the historical gold standard for methylation detection, or the targeted approach using whole genome bisulfite sequencing and Illumina BeadChip Arrays ^42,44^. ONT, however, can simultaneously capture genomic variants and measure all modifications on native DNA molecules. We identified many regions that are loosely associated with age and examined those with the largest change over age. The *PCDHG* gene cluster had the most regions that showed increased methylation fractions with age. These genes were not identified as age-related using Infinium HumanMethylation27 BeadChips targeted methylation, which is an example of how LRS-derived methylation data is more comprehensive for profiling primary tissues^57,59^. Decreases in methylation with age were less common, but similar to prior studies, we did observe a drop in methylation at *FKBP5* ^60^. By developing a methylation catalog of normal brain and prefrontal cortex tissue we are facilitating future work evaluating allele-specific mQTLs and disease-specific differential methylation patterns.

This study has several important limitations, many of which arise from the challenges of applying rapidly evolving technology at a large scale. For instance, after sequencing the NABEC cohort using R9, ONT released new flow cells and chemistries. As a result, the HBCC cohort was sequenced using R10 which offers improved read accuracy but made cross-cohort analyses more difficult. This discrepancy in sequencing platforms may affect the consistency of variant calling between cohorts, highlighting the difficulties of maintaining uniformity in large-scale projects as technology advances. Additionally, more sophisticated approaches are needed to improve SV merging methods. This is a fundamentally difficult problem because functionally equivalent SV alleles frequently vary due to independent recurrence and subsequent further acquired variation. We expect that pangenome alignment models will make it easier to group SVs by haplotypic context and features, leading to better SV sets to support downstream association analyses, and the enhancement of allele-specific methylation and expression analyses. Further, while statistical fine-mapping is an effective approach for identifying likely causal variants, functional follow-up studies are necessary to validate their biological impact and confirm their roles in gene regulation and disease mechanisms. Lastly, while this study focused on common germline variation affecting gene expression and DNA methylation in the human brain, future research will explore the role of rare variants, including population-level rare variants and those unique to individual samples, such as low-frequency somatic or mosaic variants.

In summary, we present a foundational resource comprising the first 351 brain control samples sequenced in the NIH’s Center of Alzheimer’s and Related Dementias (CARD) long-read sequencing initiative. This represents the initial phase of an ongoing effort to sequence thousands of human brains from diverse populations, including Alzheimer’s disease and related dementia (ADRD) cases and control samples. The ultimate goal of this initiative is to accelerate our understanding of the genetic and epigenetic landscape of ADRD on a global scale, providing critical insights into disease mechanisms across ancestrally diverse cohorts.

## Methods

### Ethics oversight

The NABEC study was originally approved by the Joint Addiction, Aging, and Mental Health Data Access Committee and more information can be found at the dbGaP website under the study accession ID phs001300.v4.p1. The NIH considered research using post-mortem material as nonhuman subject research and therefore no additional institutional review board approval was required. For the HBCC, brain specimens were obtained under protocols approved by the CNS IRB (NCT03092687), with the permission of the next-of-kin (NOK) through the Offices of the Chief Medical Examiners (MEOs) in the District of Columbia, Northern Virginia and Central Virginia. More information can be found at the NDA website (https://nda.nih.gov/edit_collection.html?id=3151).

### Sample Collection

For the NABEC brain samples, frozen tissues were samples from the frontal cortex for 205 neurologically normal individuals. All NABEC were obtained from three brain banks, the Banner Sun Health Research Institute(https://www.bannerhealth.com/services/research/locations/sun-health-institute/programs/body-donation/tissue), the University of Kentucky Alzheimer’s Disease Center Brain Bank (https://medicine.uky.edu/centers/sbcoa/data-sample-request) and the University of Maryland Brain and Tissue bank (https://www.medschool.umaryland.edu/btbank/). All individuals were of European ancestry and had no clinical history of neurological or cerebrovascular disease, or a diagnosis of cognitive impairment during life. Demographics, tissue source for each subject are shown in Supplementary Table S2. Average age at death was 52.67 yr (range, 15–96 yr), and 36.10% were female.

The HBCC is a brain bank within the National Institute of Mental Health (NIMH) Intramural Research Program. For the HBCC brain samples frozen tissues were dissected from the frontal cortex for 154 neurologically normal individuals. All HBCC samples were obtained from https://neurobiobank.nih.gov/ and data on this can be found at https://nda.nih.gov/edit_collection.html?id=3151 . All individuals were of African or African admixed ancestry and had no clinical history of neurological disease, or a diagnosis of cognitive impairment during life. All specimens were neurologically normal. Demographics, tissue source and for each subject are shown in Supplementary Table S2. Average age at death was 44.22 yr (range, 18–85 yr), and 36.36 % were female.

### DNA processing

The DNA processing ^61,62,63^ protocols are explained in detail and are publicly available on the protocols.io platform. In brief, ~40mg of frozen frontal cortex tissue was homogenized with a Tissueruptor instrument (Qiagen). High molecular weight (HMW) DNA was then extracted using a Kingfisher Apex instrument (Thermofisher) with a custom script and Nanobind Tissue Big DNA kit, which uses 3mm Nanobind disks (Circulomics/PacBio, US). The HMW DNA was sheared to a target size of 30kb with the DNA Fluid + needles at speed 45 for two cycles on a Megaruptor3 instrument (Diagenode).

### PromethION Long-read sequencing

For the NABEC cohort libraries were constructed using an SQK-LSK 110 kit (ONT). To ensure minimal DNA loss during library preparation, and to retain long DNA fragments, the following modifications were made to the standard SQK-LSK 110 (ONT) protocol; 1) 4.5 ug of DNA was used as starting input, 2) to reach the 4.5ug DNA input the starting DNA volume was usually higher than the recommended 47ul. In this case, the volume of AMPure XP beads was modified to match the input DNA volume, i.e. if 58ul of DNA was added then 70ul was added of AMPure XP beads, 3) during DNA repair and end-prep, 75% ethanol was used for washing rather than 70%, 4) at step 16 of DNA repair and end-prep, the original elution time was 2 minutes at room temperature. This was modified to 3 minutes at 37°C with light shaking on a Thermomixer instrument (Eppendorf) at 450rpm, 5) during Adapter ligation and clean-up, 45uL of AMPure XP beads were used 6) SFB was used, and finally 7) in step 16 of the Adapter ligation and clean-up, the final elution conditions were changed to 20 minutes at 37°C. For HBCC libraries were constructed using an SQK-LSK 114 kit (ONT)using the same modifications as above however where 2.5 ug of DNA was used as starting input.

PromethION sequencing was performed as per manufacturer’s guidelines (ONT, FLO-PRO002) with minor adjustments, such as for R.9 400ng of the library was loaded onto each primed R9.4.1 flow cell to maximize data output and for R.10 180ng of the library was loaded onto each primed R9.10.4.1 flow cell. Each sample required at least two or three loads to achieve > 115GB total data output over 72 hours.

### Basecalling and Alignment

All samples were basecalled and aligned on the NIH HPC Biowulf cluster. NABEC Basecalling was performed using Guppy v 6.12 and HBCC basecalling was performed using Guppy v 6.38.

### Small variant calling within NAPU

As a part of the NAPU workflow small variants (substitutions and indels smaller than 50bps) are called from ONT reads using either P.E.P.P.E.R-Margin-DeepVariant^64^ or DeepVariant^65,66^ (ONT_R104 model) for the R9 and R10 cohorts respectively. Small variant gVCF files, produced by DV, were then merged within cohorts using glnexus and the “DeepVariantWGS” configuration, for R9 “NoCall” gVCF entries were removed prior to merging^67^. Illumina short-read whole genome sequencing data for NABEC was obtained from dbGaP (Accession phs002636.v3.p1).

### Structural variant calling

All brain samples were processed with the NAPU workflow which uses hapdiff and Sniffles2 to produce assembly and read based structural variant calls respectively ^14,15^. Read based, reference-free, phased SVs were called using Sniffles2 v.2.3^15^. For assembly based structural variants diploid de novo assemblies are created by first making a haploid Shasta assembly and then using Hapdup v.0.12 to locally phase contigs to create a diploid assembly^14,21^. The diploid assemblies are then aligned with minimap2 (v 2.23-r1111)^68^ to the GRCh38 reference and SVs are called with Hapdiff ^14^. We used Shasta v0.11.1^21,69^ with the “Nanopore-CARD-Jan2022.conf” configuration file for R9 samples and “Nanopore-R10-Fast-Nov2022.conf” configuration with these command line arguments “--Reads.minReadLength 8000 --Align.minAlignedMarkerCount 500 --Align.minAlignedFraction 0.75” to account for the shorter read lengths observed in R10 sequenced brain samples. For samples with high read coverage we also used the “--Reads.desiredCoverage 150Gbp” which removes the shortest reads until the coverage is below the desired coverage limit. Samples sequenced with R10 had lower coverage, read lengths, and identity relative to the R9 samples, so to produce more contiguous Shasta assemblies, we relaxed the R10 length and alignment criteria (Figure 2A, 2B). We attribute the reduced quality in R10 sequence brain samples to be due to reduced quality brain tissue samples instead of due to the R10 chemistry.

SVs were merged across all samples within a cohort as well as across cohorts using either Sniffles2 or Truvari^25^. Sniffles2 was used to merge and genotype read-based SVs called by Sniffles. Truvari merge with a 75% similarity and size cutoff was used to merge hapdiff SVs within cohorts. To merge hapdiff variants, first bcftools^70^ is used to combine all vcf entries for all cohort samples which was 1.17 million NABEC SVs and 1.07 million HBCC SVs. Truvari then merged similar SVs within those sample combined VCFs which collapsed the number of NABEC SVs to 165,854 and HBCC to 210,808 SVs. After merging SVs within a cohort we used assembly alignment coverage beds created by hapdiff to then assign reference genotypes (0/0) to assembly-based SVs where there was assembly coverage but no SV called. Truvari is not currently capable of joint genotyping, so in order to include hapdiff SVs in eQTL SV analysis, which filters by genotype, we performed this manual reference genotype assignment. Sniffles2 v2.3, on the other hand, performs its own merging and joint genotyping, which resulted in 213,279 NABEC and 210,163 HBCC SVs. Sniffles2 v2.3 did take substantially longer to merge and joint genotype at over 7 and 5 hours for each cohort. Sniffles, however, is only able to merge calls it produces itself (requiring a snf file it produces during variant calling) thus, Truvari was used to merge variants between SV callers and then the two cohorts.

In order for Truvari to merge SVs from Hapdiff and Sniffles within each cohort we had to do some curation of the Hapdiff+Truvari merged cohort VCF and the Sniffles merged VCF. Truvari compares sequence similarity so the symbolic representation of Sniffles SVs was replaced with reference sequence information using a script provided by Truvari (https://github.com/ACEnglish/truvari/discussions/216). Hapdiff was able to capture the individual variation of each sample but after merging this resulted in many alleles with a small number of sample genotypes per variant. Variants with a minor allele frequency less than 5% were filtered out prior to running the QTL. For this reason we resorted to choosing Sniffles variants by setting Hapdiff quality scores to 0, since it is unable to assign a quality score to its’ assembly based SV calls, and then kept the SV representation with the highest quality score. Variants from each SV caller were combined using bcftools concat, summing to 366,717 NABEC SVs and 436,838 HBCC SVs, and after Truvari merging at 75% similarity there were 193,601 and 241,202 NABEC and HBCC cohort SVs respectively.

To ensure consistency of SV identification across both cohorts and enable comparison of allele frequencies and QTL results, we merged variants from both cohorts to create a unified set of variants. First bcftools combined samples from both cohorts and then Truvari collapse was used again to combine similar variants at 75% similarity and a max size of 60Kbp. After this merge there were 278,732 variants but over 58 thousand were still contained multi-allelic variants. These multi-allelic variants were extracted and further merged to combine larger variants over larger reference distances as well as combine insertions and deletions at the same loci. Merging these multi-allelic variants more loosely reduced those 58 thousand multi-allelic variants to just 14,717 representative SVs. These variant sets were once again combined for a final set of 234,906 single allelic variants across both cohorts and variant callers. That is 139,841 NABEC variants and 180,430 HBCC variants.

### Methylation analysis

Phased methylation beds for both R9 and R10 cohorts were created using Modkit pileup. We ran Modkit using the GRCh38 reference, restricted to 5-methylcytosine (CpG) motifs and combined across read strands. We then created aggregated regional methylation bed files using bedtools map and limiting to regions with minimum 10 CpG sites and 5 reads (https://github.com/nanoporegenomics/napu_wf/blob/r10/wdl/workflows/bedtoolsMap.wdl). We averaged methylation over CpG sites across gene promoter regions, gene bodies, and CpG Islands. We obtained the gene promoters region bed track from the Eukaryotic Promoter Database^71^. The promoter regions in the database are 60 bps for this work we extended them up and down stream by 1kb. Gene body regions were from the UCSC genome annotation database for the Dec. 2013 (GRCh38/hg38) assembly of the human genome (hg38, GRCh38 Genome Reference Consortium Human Reference 38 (GCA_000001405.15); https://hgdownload.cse.ucsc.edu/goldenpath/hg38/database/refFlat.txt.gz). To prevent multiple entries for genes with multiple isoforms the isoform with the most exons was chosen as the representative length of the gene body. CGI bedtrack is from the UCSC genome browser CpG Island track.

We used the available cohort meta-data to examine regional methylation which correlates with age in the brain. To do this we ran linear regressions on autosomal CGI’s, and whole genome windows (1kb, 50 CpG min) with post-mortem interval and gender as covariates over the sample age. After Benjamini-Hochberg multiple test correction, 28%, 145,086, whole genome windows were found to be significantly associated with age (FDR < 0.05). Of the UCSC Genome Browser Track autosomal CpG Islands 14%, 3,890, and 23%, 5,778, in NABEC and HBCC respectively were found to be significantly associated with age (FDR < 0.05).

Next we aimed to correlate methylation levels with genetic variants and performed mQTL analysis. For use in the mQTL we averaged methylation at all sites in regions of interest: promoters, gene bodies, and CpG Islands. These aggregated methylation regions were used as the input for mQTL analysis. Of the 29,598 promoters 94% NABEC and 93.5% HBCC promoters remaining after filtering for read coverage and CpG density with 27,465 (92.7%) confidently covered by both cohorts. After filtering, the NABEC and HBCC cohorts retained 93.4% and 91.4% of the 27,949 CGIs in the UCSC Genome Browser Track for a total of 90.1% of CGIs covered by both. Gene body regions were defined as 62,700 unique gene entries from Gencode V.44. After filtering these regions for coverage and minimum number of CpGs 65.3% of gene bodies were covered by both cohorts. Individually the NABEC cohort retained 67% of these gene bodies and HBCC kept 66%.

### Structural variant annotation

STIX [10.1101/2024.09.30.615931 and 10.1038/s41592–022-01423–4] was used to annotate the harmonized SV callset. STIX annotates all SVs with a long-read index, which includes 1,108 samples (1,008 samples from the 1000 Genomes Project[10.1093/nar/gkz836] and 100 samples from the HPRC project[10.1038/s41586–023-05896-x]) across 5 super-populations. We used ‘-s 100’ and ‘-T 5’ to set a padding base of 100 and required a minimum of 5 reads to define true signals. STIX long-read indices were built using excord-lr to extract raw SV signals from BAM or CRAM files. Giggle[10.1038/nmeth.4556] was used alongside STIX to create indices. All related tools for index creation and annotation can be found in the repository (https://github.com/zhengxinchang/stix-suite) with version 1.0.1. A total of 234,906 variants were annotated, of which 9,747 failed annotation because STIX does not index SV signals in random contigs. As a result, 225,159 variants were annotated successfully.

### Ancestry analysis

Ancestry predictions for 11 different ancestry groups were rendered using GenoTools^19^ ancestry pipeline with a serialized model trained on variants in common between the NeuroBooster Array^72^ and the reference panel outlined in GenoTools. This method utilizes PCA and UMAP transformations and an XGBoost classifier which targets several superpopulations and selects subpopulations relevant to Neurodegenerative diseases.

### Population level allele frequency comparison

Genotype counts from the NABEC+HBCC, NABEC, and HBCC callsets were used to calculate variant frequencies, which were compared with STIX frequency annotations^33^. Homozygous and heterozygous genotypes were collectively categorized as “mutated,” while reference genotypes (0/0) were assigned a frequency of zero. The variant frequency was defined as the proportion of mutated samples (homozygous or heterozygous) among the total number of samples with valid genotypes (excluding missing genotypes, ./.). To ensure robust frequency estimates, minimum genotype thresholds were applied: 350 samples for NABEC+HBCC, 200 samples for NABEC, and 140 samples for HBCC. Pearson correlation coefficients were calculated to evaluate the concordance between STIX annotations and variant frequencies observed in the respective callsets.

### Expression analysis

#### NABEC Bulk short-read RNA sequencing data

To identify the functional impact of the new genetic variants, sample data was taken from the North American Brain Expression Consortium (NABEC; dbGaP Accession phs001300.v4.p1). Bulk RNA-seq data from all samples was quantified using Salmon ^73^ with the filtered Gencode v43 human transcriptome index. Bulk frontal cortex expression from NABEC contained 206 samples covering 60880 quantified genes. Sex specific genes were subsetted and plotted to identify any sample gender mismatches, and for brain samples. 34102 genes with a missingness over 0.25 were excluded, leaving 26778 well detected genes. To standardize, expression data was quantile transformed and were scaled to 0 to 1.

#### HBCC Bulk short-read RNA sequencing data

To identify the functional impact of the new genetic variants, sample data was taken from the HBCC, https://nda.nih.gov/edit_collection.html?id=3151). Bulk RNA-seq data from all samples was quantified using Salmon ^73^ with the filtered Gencode v32 human transcriptome index. Bulk frontal cortex expression from HBCC contained 86 samples covering 60880 quantified genes. Sex specific genes were subsetted and plotted to identify any sample gender mismatches. 34,201 genes with a missingness over 0.25 were excluded, leaving 26679 well detected genes.

### Quantitative trait loci analysis

The expression and methylation data was normalized by quantile-normalization and min-max scaled and was used for cis quantitative trait loci (QTL) analysis using tensorQTL.^74^ Sex, age, genetic PC, trait PC, postmortem interval, group in the datasets (‘SH’, ‘UKY’, ‘UMARY for NABEC) were used as covariates. QTL analyses were analyzed using three variant sets including harmonized SVs, SNVs (illumina data in NABEC, deepvariant called data in HBCC), and harmonized SV plus SNV. Genetic PCs were calculated for each variant type and caller. Variants that meet the following criteria have been removed:; 1) minor allele frequency < 0.05, 2) genotyping rate < 0.95, 3) Hardy-Weinberg equilibrium pvalue > 0.001, 4) variants not located chromosome 1–22. Genetic principal components (PCs) were generated in PLINK (v1.9). PCs of gene expression data were calculated using the PCA module from the sklearn.decomposition package. The number of genetic PCs and expression PCs to include in the QTL analysis was selected by determining the point of maximum curvature using kneed (https://github.com/arvkevi/kneed). All variant–phenotype pairs within a specified window (± 1 Mb) around the phenotype were calculated.For multiple correction, we used q-value and defined false discovery rate smaller than 0.05 as significant.

### Fine-mapping of causal variants QTL

We applied CAVIAR to assess LD and identify the most likely causal variant for each eQTL and mQTL. CAVIAR integrates summary statistics with LD information across the associated locus to estimate the causal probability of each variant. QTL were identified with qvalue < 0.05. Z-scores were computed for each gene-variant pair using linear regression slope and standard deviation as an input for CAVIAR. For each significant loci, we used Z-scores and LD for the top 100 variants including the best SV (if no SV was included in the top 100 variants) with causal set size of 1 to run CAVIAR. Posterior probabilities were used to evaluate the causality of each variant.^75^

### Short-read SV calling for QTL comparison

To assess whether the LRS QTL hits identified in this study could also be detected using SRS methods, we analyzed existing SRS-derived SV calls for the NABEC cohort, as previously described by Billingsley et al^76^. In brief, SV data were generated from WGS processed through the AMP-PD pipeline. DNA samples were sequenced by either Macrogen or the Uniformed Services University of the Health Sciences (USUHS) using Illumina TruSeq DNA library preparation protocols, resulting in average insert sizes of ~300–410 bp. Sequencing was conducted on the Illumina HiSeq X platform, and reads were aligned to the GRCh38DH reference genome using the Broad Institute’s standardized functional equivalence pipeline. SVs were identified using the GATK-SV pipeline (https://github.com/broadinstitute/gatk-sv), which integrates multiple SV callers, depth-based analyses, and downstream filtering to ensure high-quality variant discovery. In total 353,426 SRS-derived SV calls were available for 189 NABEC samples.

## Supplementary Material

Supplement 1

Supplement 2

Supplement 3

## Data Availability

Human brain sequencing datasets are under controlled access and require a dbGap application (phs001300.v4) (phs000979.v4). Afterwards, the data will be available through the restricted AnVIL workspace.
