# Supplementary material for: Long-read sequencing of hundreds of diverse brains provides insight into the impact of structural variation on gene expression and DNA methylation
Source: bioRxiv. 2024 Dec 17:2024.12.16.628723. Preprint. [Version 1] doi: 10.1101/2024.12.16.628723 (PMC11702628; doi:10.1101/2024.12.16.628723)
Supplement: Supplement 3 [file NIHPP2024.12.16.628723v1-supplement-3.pdf]

bioRxiv preprint doi: https://doi.org/10.1101/2024.12.16.628723; this version posted December 18, 2024. The copyright holder for this preprint (which was not certified by peer review) is the author/funder. This article is a US Government work. It is not subject to copyright under 17 USC 105 and is also made available for use under a CC0 license.

Supplementary Figure Legends:

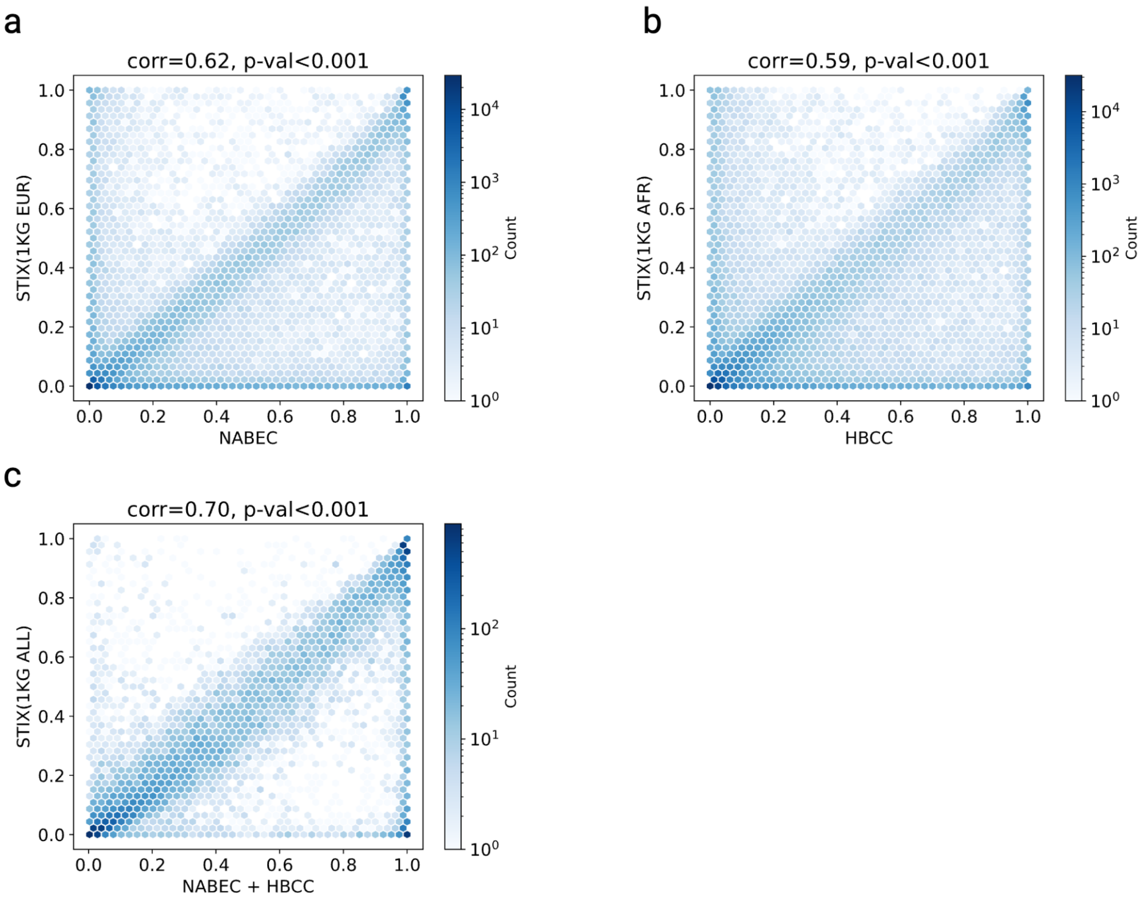

Supplementary Figure 1. Comparison of STIX frequency and variant frequency within three callsets. Variants with valid genotypes were included. a) NABEC only, minimal genotypes = 200. b) HBCC only, minimal genotypes = 140. c) NABEC + HBCC, minimal genotypes = 350. Pearson correlation was used to evaluate the concordance between the STIX frequency and variant frequency within the callsets. The

bioRxiv preprint doi: https://doi.org/10.1101/2024.12.16.628723; this version posted December 18, 2024. The copyright holder for this preprint (which was not certified by peer review) is the author/funder. This article is a US Government work. It is not subject to copyright under 17 USC 105 and is also made available for use under a CC0 license.

color indicates the number of variants located in specific hexagonal bins.

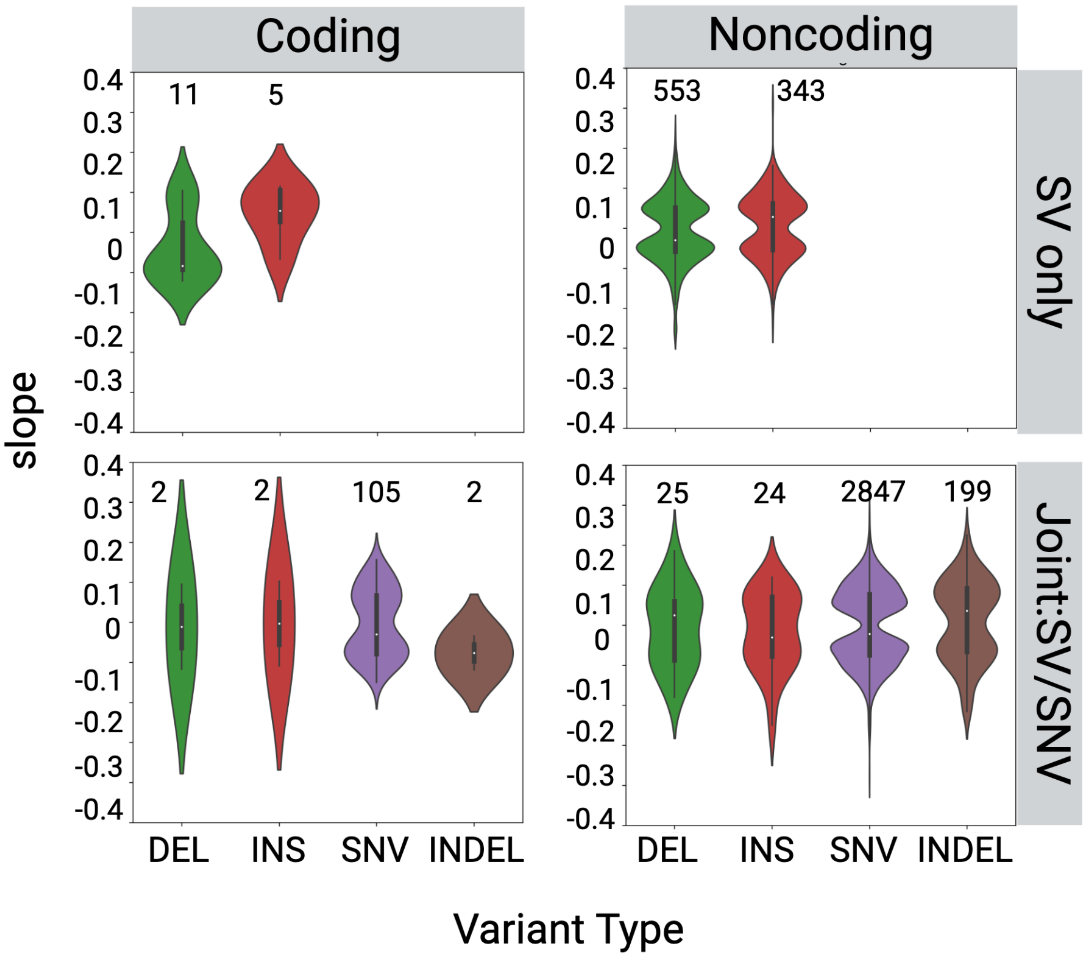

Supplementary Figure 2. Comparison of effect sizes between variant types for eQTL, left panel. In each panel, panels is divided in four sections, top left SV-eQTL hits in overlapping with coding regions, top right SV-eQTL hits in non-coding regions, bottom left SV-SNV joint eQTL hits in coding regions, bottom right SV-SNV joint eQTL hits in non-coding regions.

bioRxiv preprint doi: https://doi.org/10.1101/2024.12.16.628723; this version posted December 18, 2024. The copyright holder for this preprint (which was not certified by peer review) is the author/funder. This article is a US Government work. It is not subject to copyright under 17 USC 105 and is also made available for use under a CC0 license.

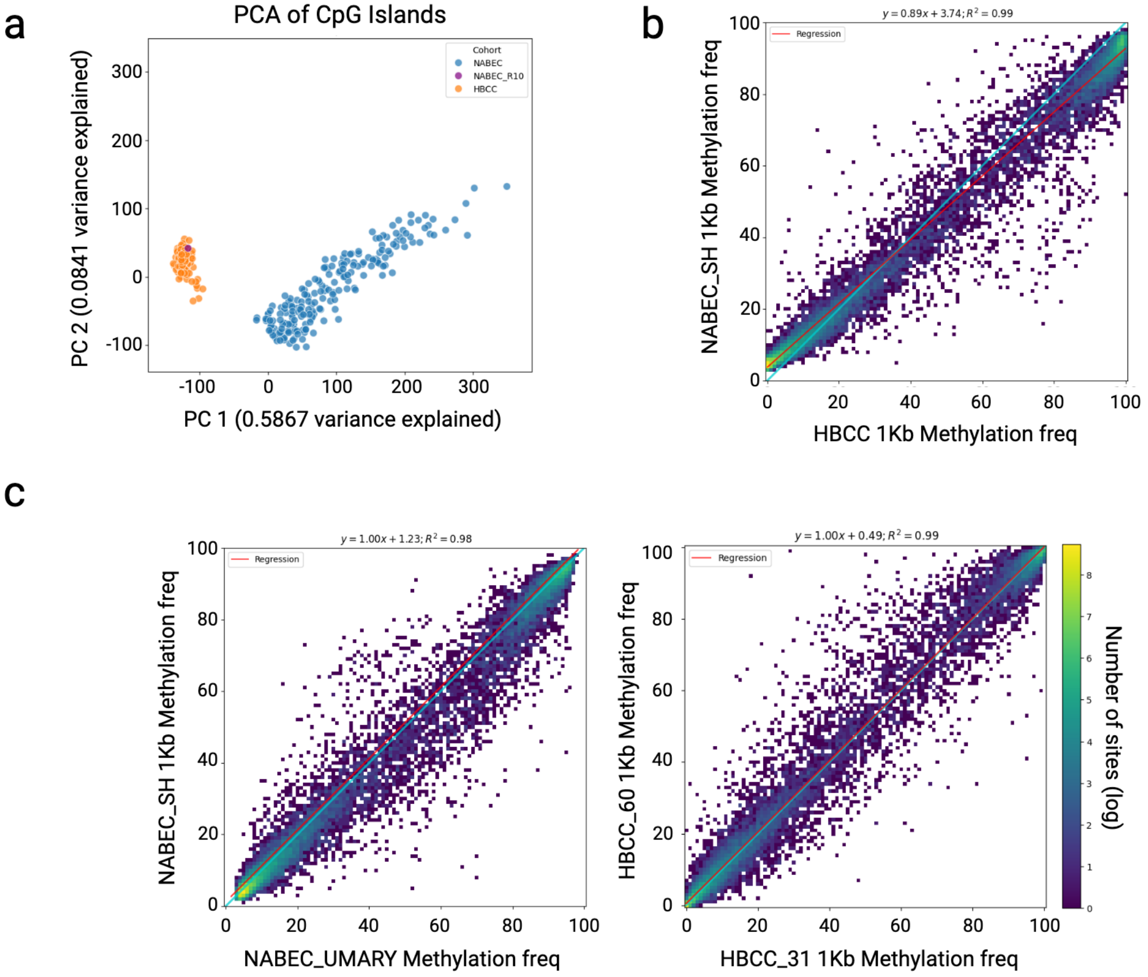

Supplementary Figure 3. a. PCA of NABEC and HBCC methylation averaged over CpG Islands. One NABEC sample was sequenced with both R9, plotted in blue, and R10, purple. The clustering of the R10 sequenced European NABEC sample with the other R10 sequenced African or African admixed ancestry HBCC samples suggests that the technical differences in methylation frequency are stronger than the ancestry differences. b,c. Heatmaps of pairwise comparisons of autosomal methylation frequency between a NABEC and HBCC sample (left) two NABEC samples, and two HBCC samples. The light green line is plotted on the diagonal and the regression line fitted to the data is plotted in red. In the far left panel, the red line is off the diagonal with respect to the NABEC cohort (y-axis); the regression line equation and the R^2 correlation are the titles of these panels.

bioRxiv preprint doi: https://doi.org/10.1101/2024.12.16.628723; this version posted December 18, 2024. The copyright holder for this preprint (which was not certified by peer review) is the author/funder. This article is a US Government work. It is not subject to copyright under 17 USC 105 and is also made available for use under a CC0 license.

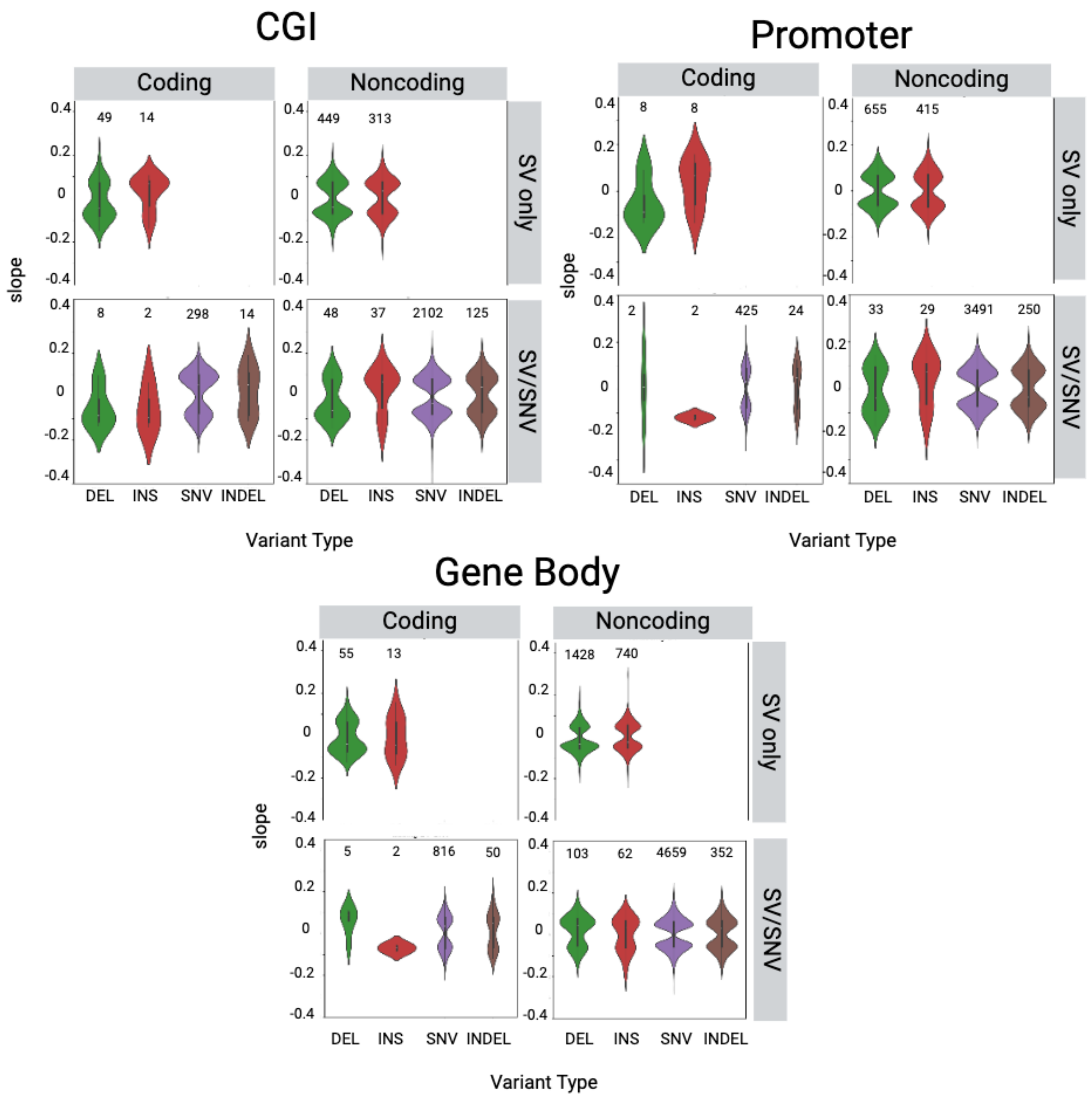

Supplementary Figure 4. Comparison of effect sizes between variant types for mQTL, left panel; Cpg islands, center panel; promoters, right panel; gene bodies. In each panel, panels is divided in four sections, top left SV-mQTL hits in overlapping with coding regions, top right SV-mQTL hits in non-coding regions, bottom left SV-SNV joint mQTL hits in coding regions, bottom right SV-SNV joint mQTL hits in non-coding regions.
